# Effect of immediate kangaroo mother care (iKMC) on neonatal mortality and culture-positive sepsis in low-birth-weight neonates in district hospitals in Chhattisgarh, India (PRISM study): protocol for a stepped-wedge cluster randomized trial

**DOI:** 10.1186/s13063-025-09083-3

**Published:** 2025-10-09

**Authors:** Satya Prakash, Atul Jindal, Kajal Jain, Rohit Anand, Vivek Kumar, Anudita Bhargava, Sarita Mohapatra, Nitya Wadhwa, Ramesh Agarwal, M. Jeeva Sankar

**Affiliations:** 1 All India Institute of Medical Sciences, New Delhi, India; 2 All India Institute of Medical Sciences, Raipur, Chhattisgarh India; 3 Clinical Development Services Agency, BRIC-Translational Health Science and Technology Institute, Faridabad, Haryana India

**Keywords:** Immediate kangaroo mother care (iKMC), Low-birth-weight neonates, Neonatal sepsis, District hospitals

## Abstract

**Background:**

The high rates of culture-positive sepsis, sepsis-related mortality, and multidrug resistance in neonates admitted to special care newborn units in district hospitals (DH) in India necessitate urgent actions to prevent infections in these settings. Immediate kangaroo mother care (iKMC), initiated before stabilization within the first few hours of life, has been shown to reduce neonatal mortality among low birth weight (LBW) neonates admitted to tertiary care hospitals. However, the effect of iKMC on sepsis and sepsis-related mortality, particularly in DH, remains unclear. This study aims to evaluate whether iKMC can lower the incidence of mortality or culture-positive sepsis in LBW neonates admitted to neonatal units in district hospitals.

**Methods:**

This stepped-wedge cluster randomized trial will be conducted in ten district hospitals in Chhattisgarh, India, over 42 months. All neonates weighing between 1000 and 1799 g at birth and admitted to the participating hospitals, in whom KMC can be initiated within 12 h of life, will be eligible for inclusion. During the pre-intervention period (control), routine KMC will be practiced at the sites as is. Following a baseline period of 6 months, iKMC will be implemented sequentially in ten steps across the ten study sites at intervals of 3 months. The intervention includes initiating continuous skin-to-skin contact with the mother or relatives within 12 h of life, continuing KMC until discharge or weight 2.5 kg, and providing breastfeeding support. The primary outcome is the incidence of all-cause neonatal mortality and/or culture-positive sepsis until 28 days of life. Relative risks with 95% confidence intervals will be reported and analyzed using a generalized linear mixed model. An intention-to-treat analysis will be conducted.

**Discussion:**

The PRISM (Protective Role of Immediate KMC in neonatal Sepsis and Mortality) trial will sequentially implement iKMC for LBW neonates in ten district hospitals with a relatively high burden of sepsis in India. If proven effective in reducing the risks of sepsis or neonatal mortality, the trial will provide necessary evidence for its safety and efficacy, as well as the impetus for implementing and upscaling the intervention in district hospitals across India and other low- and middle-income countries.

**Trial registration:**

Clinical Trial Registry of India, CTRI/2024/09/074269. Registered on 24/09/2024, https://ctri.nic.in/Clinicaltrials/pmaindet2.php?EncHid=MTE0MzY3&Enc=&userName=immediate%20kangaroo%20mother%20care.

**Supplementary Information:**

The online version contains supplementary material available at 10.1186/s13063-025-09083-3.

## Background

Globally, around 20 million low-birth weight (LBW) neonates are born each year, constituting around 15% of all births. In Southern Asia alone, 8.8 million LBW neonates are born annually, with 26% of all neonates being LBW [1]. Around 80% of all neonatal deaths occur in LBW neonates; a vast majority of these occur in Southern Asia and sub-Saharan Africa [2]. Over half of these deaths are attributable to prematurity and neonatal infections [3].

A recent multicentric study aimed at evaluating the burden of neonatal sepsis in district hospitals in India reported a high incidence of culture-positive sepsis (3.2%, 95% CI 0.6–14.4%), with a case fatality rate of 36.6% (78/213; 95% CI 12.1–71·0) in neonates admitted to the special care newborn units (SCNU) of these hospitals [4]. Alarmingly, around 80% of the common pathogens were multi-drug resistant. The high rates of sepsis, case fatality, and sepsis-related mortality in low- and middle-income countries (LMIC) highlight an urgent need to strengthen infection control practices and identify newer simple and effective interventions for sepsis prevention.

Kangaroo mother care (KMC), i.e., continuous skin-to-skin contact of preterm LBW neonates with their mothers’ chest and feeding exclusively with breast milk, is one of the most effective interventions for preventing neonatal mortality [5]. Traditionally, KMC is initiated in LBW infants after stabilization, which delays the initiation of KMC in most preterm neonates to around 3–24 days of life [6]. Given that around 45% of all neonatal deaths occur on the first day of life, and 80% occur within the first week, most deaths in LBW neonates occur before KMC can be initiated [7]. To extend the benefits of KMC to the first few days of life, a few recent studies have examined the role of early-initiated KMC (or immediate KMC), initiated as soon as possible after birth irrespective of clinical stability, on neonatal outcomes in hospital settings [8–10]. In a meta-analysis comparing early- versus late-initiated KMC, early-initiated KMC showed a significant reduction in mortality by day 28 of life (RR 0.78, 95% CI 0.66 to 0.92; 3 trials, 3533 infants, high certainty evidence) [6].

Several mechanisms have been proposed to explain a possible protective effect of iKMC on neonatal sepsis, including early exposure to protective maternal microflora, reduced cross-infection due to limited handling by healthcare workers, and improved breastmilk feeding [11]. The investigators of the WHO iKMC trial performed a post-hoc analysis to assess the effect of iKMC on neonatal sepsis in LBW neonates after 48 h of life. There was a significant reduction in clinically suspected sepsis (RR 0.86, 95% CI 0.75–0.99) and sepsis-related mortality (RR 0.63, 95% CI 0.47–0.85) in the iKMC arm compared to the control arm. However, the incidence of culture-positive sepsis did not differ between the two arms (8.7% vs 8.4%) [11]. In this trial, blood culture facilities were unavailable at all study sites, and the unblinded nature of the intervention raised concerns about potential bias in the clinical suspicion of sepsis. Consequently, the effect of iKMC on confirmed sepsis could not be thoroughly assessed.

Due to significant deficiencies in health infrastructure and referral facilities, most LBW neonates in LMICs are managed in secondary-level health facilities, which are often poorly equipped [12, 13]. The efficacy, safety, and feasibility of implementing iKMC in these facilities, which have a high baseline incidence of sepsis and case-fatality rates, remain unclear. The PRISM (Protective Role of Immediate KMC in neonatal Sepsis and Mortality) study aims to evaluate whether iKMC can reduce the incidence of culture-positive sepsis and/or mortality in LBW neonates admitted to neonatal units in district hospitals.

## Methods

This manuscript has been prepared according to the Standard Protocol Items: Recommendations for Interventional Trials (SPIRIT) statement [14].

### Objectives and trial design

#### Research question

Among low birth weight neonates (1000–1799 g at birth) admitted within 12 h of life to the special care newborn units (SNCU) of district hospitals (P), does immediate kangaroo mother care (iKMC) initiated within 12 h of life (I) reduce the incidence of all-cause neonatal mortality or culture-positive sepsis (O) compared to conventional care (C), i.e., care in a radiant warmer until their condition stabilizes, followed by the initiation of KMC?

#### Objectives

##### Primary

To evaluate the effect of immediate kangaroo mother care (iKMC) on the incidence of all-cause neonatal mortality and/or culture-positive sepsis in neonates with a birth weight of 1000–1799 g who are admitted to the special newborn care units of district hospitals in Chhattisgarh, India.

##### Secondary

To evaluate the effect of immediate kangaroo mother care (iKMC) on:

- All-cause neonatal mortality
- Culture-positive sepsis in the first 28 days of life
- Time to neonatal mortality
- Early (< 7 days of life) neonatal mortality
- Incidence of early-onset (< 72 h of life) and late-onset (≥ 72 h of life) culture-positive sepsis
- Incidence of clinical sepsis
- Exclusive expressed breast milk feeding or exclusive direct breastfeeding at discharge
- Hospital-free days
- Pathogen profile and antimicrobial resistance pattern in neonates with culture-positive sepsis
- Skin and gut colonization of the neonates at day 3 (± 24 h) and day 7 (± 24 h)

Supplementary Table 1 provides the sepsis-related definitions used in the study.

*Study design:* Stepped-wedge cluster randomized controlled trial.

*Setting:* Special newborn care unit (SNCU) of 10 district hospitals in Chhattisgarh, India.

Infants weighing less than 1.8 kg are regularly separated from their mothers immediately after birth and admitted to the SNCUs. The care in SNCUs includes maintaining warmth, feeding expressed breast milk, and administering intravenous fluids, antibiotics, oxygen, and continuous positive airway pressure (CPAP) if necessary. More advanced treatments like surfactant therapy and mechanical ventilation are generally unavailable in most SNCUs. KMC is commonly practiced and typically starts after stabilization, usually around 3 to 7 days after birth.

**Fig. 1:**
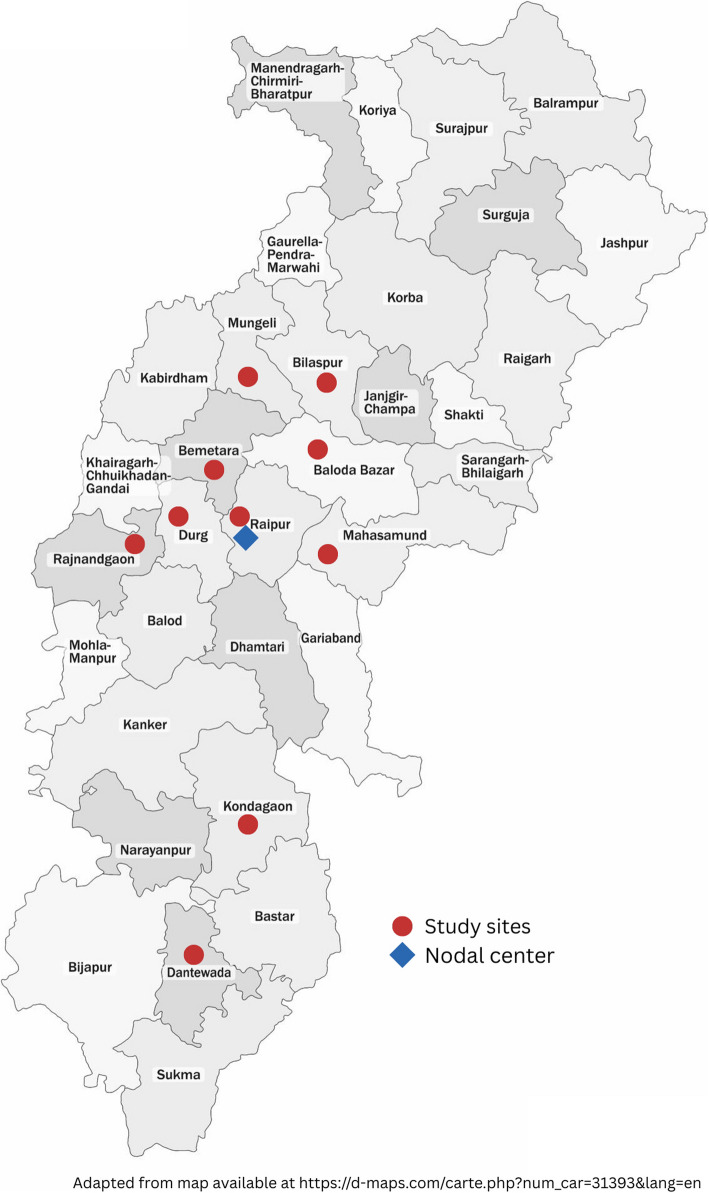
Map of Chhattisgarh showing the location of the study sites and the nodal center

*Selection of clinical sites*: Out of 28 SNCUs in Chhattisgarh, 10 were selected based on the following characteristics: space available for setting up a Mother-Newborn Care Unit (MNCU), the number of admissions of neonates with birth weight less than 1.8 kg in a month, the distance from AIIMS Raipur (nodal center), and the feasibility of transporting blood culture samples to AIIMS Raipur. Figure 1 depicts a map of Chhattisgarh showing the location of the study sites.

*Study duration:* 42 months.

##### Inclusion criteria

All live low birth weight (1000–1799 g) neonates admitted to the study hospitals, irrespective of place of birth, in whom KMC can be initiated within 12 h of life.

##### Exclusion criteria 

1. Neonates born to mothers who are either unwilling to give consent or are ill and unlikely to be able to provide KMC within the first 3 days after birth.
2. Neonates born to mothers younger than 18 years old.
3. Triplets or higher-order births.
4. Major congenital malformation that interferes with the intervention, or the intervention interferes with the required care for the congenital malformation.
5. Neonates requiring intubation and mechanical ventilation or inotropes for shock within the first 12 h of life.
6. If the mother-infant pair cannot be enrolled within 12 h of the neonate’s birth for any reason (such as if the mother is admitted elsewhere/deceased).

### Sites randomization and participant enrollment

**Fig. 2:**
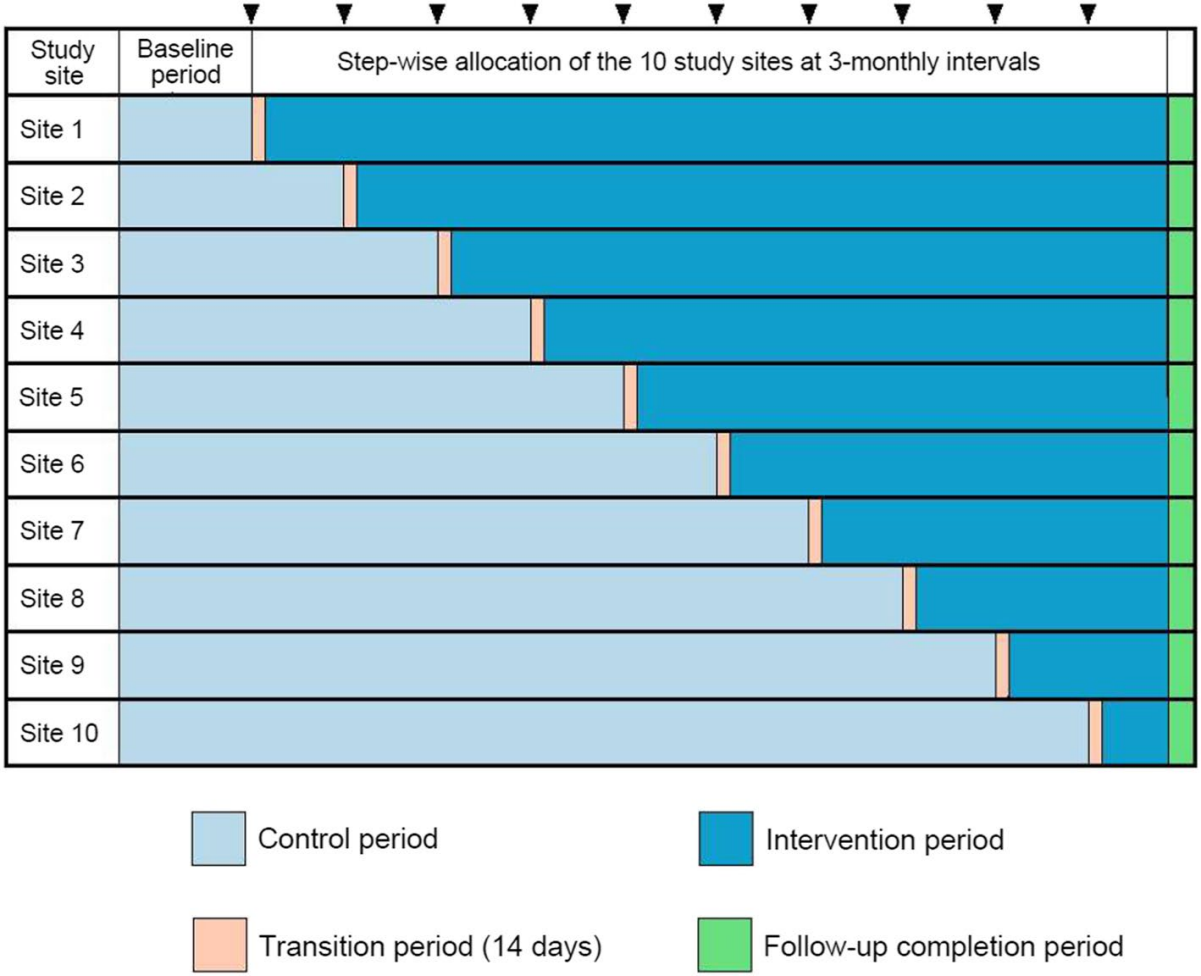
The stepped wedge design for the study

In a stepped-wedge study, the intervention is gradually implemented in steps at the participating clinical sites. The order of implementing the intervention will be determined using a computer-generated random sequence placed in sequentially numbered, opaque sealed envelopes at the coordinating center. We will have a baseline period of 6 months before randomizing the first site. The subsequent study period will be divided into ten phases, each lasting 3 months. Before each step, the envelope will be opened, and the clinical site randomly selected for that phase will be notified about initiating iKMC and ensuring care for the neonate in the mother-newborn care unit (MNCU). A 5- to 7-day training session will be conducted for the research staff prior to rolling out the intervention. Therefore, the clinical sites will be informed about the initiation of the intervention at their site 14 days before the scheduled phase. These 14 days, starting after 3 months of the intervention period completed at the previous site, will be referred to as the “transition period.” The intervention period will begin once the transition period is over and will continue for 3 months before the next site is randomly selected for the intervention. All ten clinical sites will have received the intervention by the end of the ten steps. Thus, each site will have its pre-intervention period as the control period (Fig. 2).

### Sample size calculation

Based on preliminary data collected from the study sites, we found a 27% incidence of mortality or culture-positive sepsis in neonates with birth weights of 1–1.799 kg. Assuming a 22% relative reduction in its incidence in the intervention arm (i.e., 21.1% mortality or culture-positive sepsis), variable cluster sizes centered around a mean rate of 36 neonates per 3-month phase, an intra-cluster correlation of 0.01, ten phases preceded by a baseline period of 6 months, 14-day transition periods, and a one-sided alpha of 0.025, we calculate a power of 92% with an average total sample size of 4920.

Following the baseline period, we plan to review control incidence rates to re-evaluate the sample size and study duration. Additionally, the final sample size may be lower if the adaptive trial stops early for futility or success.

### Study sites

The SNCUs to be included in the study are in the following district hospitals of Chhattisgarh: Bilaspur, Bemetara, Dantewada, Durg, Kondagaon, Mahasamund, Raipur, Rajnandgaon, Mungeli, and Balodabazar (Fig. 1). All the SNCUs are within 30 to 300 km from AIIMS, Raipur. All the microbiology work will be done at AIIMS, Raipur.

### Blinding

Due to the nature of the intervention, neither the healthcare providers involved in the care of the neonates nor the outcome assessors (research staff) can be blinded, as they can observe whether the newborn is in the SNCU or the MNCU with the mother. The data analysts will be unaware of the allocation periods to minimize bias.

### Participants: screening and enrollment

The healthcare staff of the hospital or the research nurse (RN) will weigh every neonate born in the hospital or admitted to the SNCU. After weighing, the RN will assess the mother-newborn dyad’s eligibility for the study. Mothers will be eligible for enrollment in this study if they are at least 18 years of age and their consent is confirmed by the guardian (parent or husband). If the mother and neonate are eligible, neonates will be enrolled after obtaining informed written consent from the mother/guardian.

To ensure timely enrollment and initiation of intervention in the eligible inborn neonates, pregnant women at risk of delivering an LBW neonate will be pre-screened prior to delivery. This includes pregnant women with anticipated preterm delivery, antenatally suspected intrauterine growth restriction (based on fundal assessment or obstetric ultrasound), multiple gestation, hypertensive disorders of pregnancy, and severe anemia. These pregnant women will be approached by the RN, informed about the trial, and invited to participate in the trial. Those women who are in advanced stages of labor will not be approached. After delivery, eligibility and consent will be reaffirmed.

### Control and intervention

After enrollment, all neonates enrolled during the control period will receive standard care, following the usual practices at the sites, which involve separating the mother and infant and admitting them to the SNCU until the neonate achieves clinical stability. Besides iKMC and the care of the neonate in the MNCU, all other interventions will remain consistent across both intervention and control groups. Feeding will begin based on the neonate’s clinical condition, with expressed breast milk initially administered through an orogastric tube or cup/*paladai*, transitioning to direct breastfeeding when the neonate is deemed ready. Brief KMC sessions will start in the control period once the neonates are stable, typically after meeting specific criteria such as being off continuous positive airway pressure (CPAP), requiring less than 30% oxygen, and tolerating partial enteral feeds, usually at least 24 h old. The mother will visit the SNCU to provide these short KMC sessions several times a day during feeding times. Continuous KMC will be initiated in the control period once the neonates meet stability criteria for at least 24 h and can be transferred to the KMC ward.

The routine KMC will involve the following three main components:

- Skin-to-skin contact (SSC): The neonate will be placed directly on the mother’s chest after the initial stabilization.
- Exclusive breastfeeding: The neonate is encouraged to breastfeed exclusively.
- Supportive Care: The mother provides continuous care and comfort to the neonate, including holding, comforting, and promptly responding to the neonate’s needs.

KMC will preferably be provided by the mother. In her absence, any other female family member (surrogate) designated by the family can also provide KMC. The hours of KMC offered by the mother or any other family member will be recorded. KMC will continue until the discharge of the neonate or until they reach a weight of 2500 g, whichever comes first.

In the intervention period, all newborns enrolled will receive iKMC and regular preterm care as in the control period. iKMC is immediate and continuous KMC, which means it will be started soon after birth (within 12 h of life) and given continuously—for at least 16 h per day—before and after stabilization until discharge or the neonate achieves 2500 g weight, whichever is earlier.

Thus, the iKMC will consist of the following additional points:

1. Skin-to-skin contact: The implementation research nurse will assist the mother or surrogate in starting iKMC by placing the newborn directly on the mother’s chest. This will be done as soon as possible after delivery or admission, whether in the delivery room, operating theatre, or upon SNCU admission, and will continue during transfer and MNCU stay. Monitoring of oxygen saturation and heart rate will be performed using a pulse oximeter. The implementation research nurse will also help transfer the mother (or surrogate) and the neonate to the MNCU in SSC. She will further support the mother or surrogate in providing continuous KMC after the neonate is admitted to the MNCU.
  The mother and infant will remain in the MNCU until the infant meets the stability criteria. These criteria include breathing spontaneously without oxygen or CPAP support, maintaining oxygen saturation above 90% in room air, having no apnea episodes, a heart rate between 80 and 180 beats per minute, an axillary temperature between 36.0 and 37.4 °C, and not requiring intravenous fluids. Once the infant stabilizes, they will be transferred from the MNCU to the KMC ward. Continuous KMC will be provided until they are ready for discharge. We anticipate that a separate space for a KMC ward may not be available at all study sites. If a KMC ward is unavailable, the MNCU ward space will serve as the KMC ward during the pre-intervention period. In the intervention period, designated maternal beds within the MNCU ward will be allocated as step-down beds, which will act as KMC ward beds.
  If the infant requires a procedure or treatment that cannot be performed during SSC, they will be temporarily moved to the radiant warmer. SSC will be paused during this time, but it will resume as soon as the procedure or treatment is completed. If a mother is unavailable for an extended period, a surrogate caregiver will continue SSC until the mother can take over again.
2. Supportive care: The MNCU will feature separate beds for mothers next to their infants’ radiant warmers. Obstetric staff will deliver medical care to the mothers admitted to the MNCU. Mothers will receive support and encouragement to initiate and maintain early and exclusive breastfeeding. In the MNCU, assistance will be available to mothers as they attempt to breastfeed their infants. Even if direct breastfeeding is not possible, placing the infant at the breast allows for practice in attachment and suckling. When feasible, mothers will be advised to express and feed colostrum early.
  All routine care will be provided during SSC. The outcome assessor research nurse team will record any interruptions in SSC to determine the daily duration of the intervention. The implementation research nurse will also assist the mother in expressing early milk and help the neonate with breastfeeding.

#### Care of the mothers

Mothers enrolled during the control period will receive regular postnatal care in the postnatal ward until their newborn is ready to be transferred to the KMC unit, after which they will also be moved to the KMC ward.

During the intervention period, mothers will be transferred to the MNCU along with their newborns. Mothers who are unstable or need specialized care will not be moved to the MNCU; instead, they will remain in the labor room or postnatal ward according to standard hospital protocols. These mothers will only be shifted to the MNCU for KMC once they no longer require special care. Obstetric staff will continue to monitor and care for all mothers after childbirth.

### Study endpoints

**Fig. 3:**
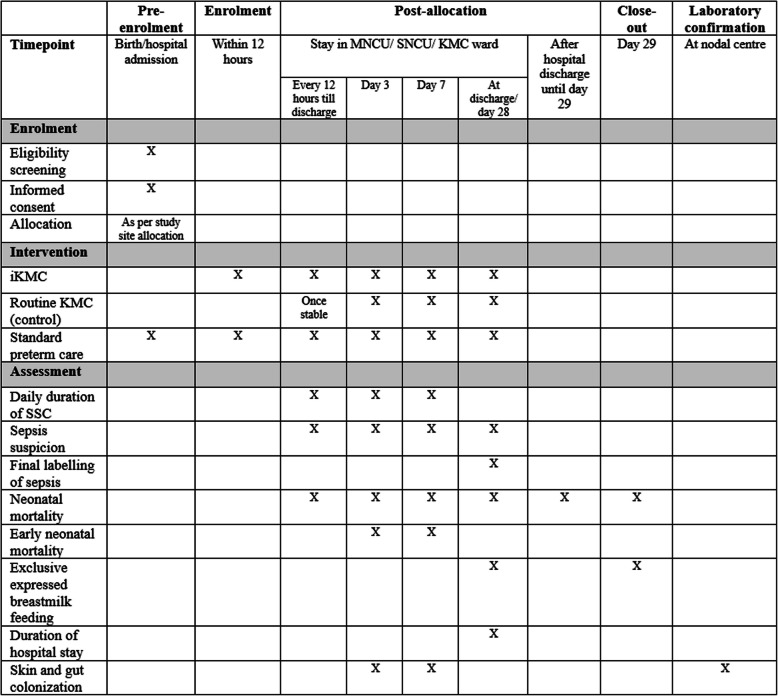
Trial schedule of enrollment, interventions, and assessments. Abbreviations: MNCU mother newborn care unit, SNCU special newborn care unit, KMC kangaroo mother care, SSC skin-to-skin contact

Table 1 depicts the study objectives and corresponding endpoints, and Fig. 3 shows the schedule of outcome assessment.

**Table 1:** Study objectives and corresponding endpoints

*Objective*	*Endpoint*
**Primary**	
To evaluate the effect of immediate kangaroo mother care (iKMC) on the incidence of all-cause neonatal mortality and/or culture-positive sepsis in neonates with birth weights of 1000–1799 g and admitted to level 2 units of the district hospitals	• All-cause neonatal mortality and/or culture-positive sepsis until 28 days of life
**Secondary**	
To evaluate the effect of immediate kangaroo mother care (iKMC) on the incidence of all-cause neonatal mortality in neonates with birth weights of 1000–1799 g and admitted to level 2 units of the district hospitals	• All-cause neonatal mortality until 28 days of life
To evaluate the effect of immediate kangaroo mother care (iKMC) on the incidence of culture-positive sepsis in neonates with birth weights of 1000–1799 g and admitted to level 2 units of the district hospitals	• Culture-positive sepsis until 28 days of life
To evaluate the effect of iKMC on time to neonatal mortality	• Time to all-cause neonatal mortality, using Kaplan–Meier survival estimates
To evaluate the effect of iKMC on early neonatal mortality	• All-cause neonatal mortality within first 7 days of life
To evaluate the effect of iKMC on the incidence of early-onset and late-onset culture-positive sepsis	• Early**-**onset (< 72 h of life) culture-positive sepsis • Late onset (≥ 72 h of life) culture-positive sepsis • Time to culture-positive sepsis, using Kaplan–Meier survival estimates
To evaluate the effect of iKMC on incidence of clinical sepsis	• Clinical sepsis in neonates within 28 days of life
To evaluate the effect of iKMC on exclusive expressed breast milk feeding or exclusive direct breastfeeding at discharge	• Exclusive expressed breast milk feeding or exclusive direct breastfeeding at discharge
To evaluate the effect of iKMC on the number of hospital-free days	• Number of hospital-free days, defined as the number of days, up to 28 days of life, not admitted to the hospital
**Exploratory**	
To describe the pathogen profile and antimicrobial resistance pattern in neonates with culture-positive sepsis	• Number of organisms isolated from neonates • Organisms with resistance to various antimicrobial agents
To describe the skin and gut colonization of the neonates at day 3 (± 24 h) and day 7 (± 24 h) in the two periods	• Alpha and beta diversity of the organisms colonizing skin and gut at day 3 • Alpha and beta diversity of the organisms colonizing skin and gut at day 7

### Monitoring and sample collection

#### Monitoring of enrolled newborns

The research nurses will be divided into two teams—the implementation team and the outcome assessor team. The implementation team shall be responsible for enrolling neonates and supporting mothers in providing continuous skin-to-skin contact in the MNCU. However, they will not participate in routine newborn care provided by the regular nursing and medical staff employed at the SNCUs. The outcome assessor team will monitor the enrolled neonates for the specified outcomes during their stay in the SNCU/MNCU/KMC and record the information regarding vital parameters every 12 h up to hospital discharge/death. In neonates with suspected sepsis (based on pre-specified criteria), sepsis workup, including cultures of blood and other body fluids, will be performed by the outcome assessor team with the support of the clinical team as per the Standard Operating Procedures (SOPs).

#### Sample collection and processing

Blood culture samples (1 mL) will be collected from the eligible neonates in BacT/ALERT bottles at the suspicion of sepsis. The bottles with samples will be kept in an incubator at 37 °C and transported as early as possible (within 24 h of sample collection). The field worker from the SNCU site will transport the samples (in a carrier bag) to AIIMS Raipur. The samples collected at AIIMS, Raipur, will be processed at the microbiology laboratory. Samples positive for bacterial growth will be sub-cultured in 5% sheep blood agar (BioMerieux, Marcy l’Etoile, France) and MacConkey agar (Oxoid, Hampshire, UK). Identification of isolates and antimicrobial susceptibility testing will be performed using the automated (VITEK 2) system, as per the Clinical and Laboratory Standards Institute (CLSI) guidelines. Blood culture bottles will be incubated in an automated culture machine for at least 5 days before being labeled sterile.

#### Microbiome sample collection

In each period from three randomly selected sites, sample collection (skin swabs and fecal samples) from the neonates will be done on day 3 (± 24 h) and day 7 (± 24 h) after enrollment. For a neonate, four samples will be collected. In each period, 30 neonates will be sampled from three sites. 16S rRNA sequencing will be done to understand the microbiome in a sample from the neonate. Alpha- and beta-diversity will be calculated to understand the microbial diversity within a sample and between the samples collected during two periods.

**Fig. 4:**
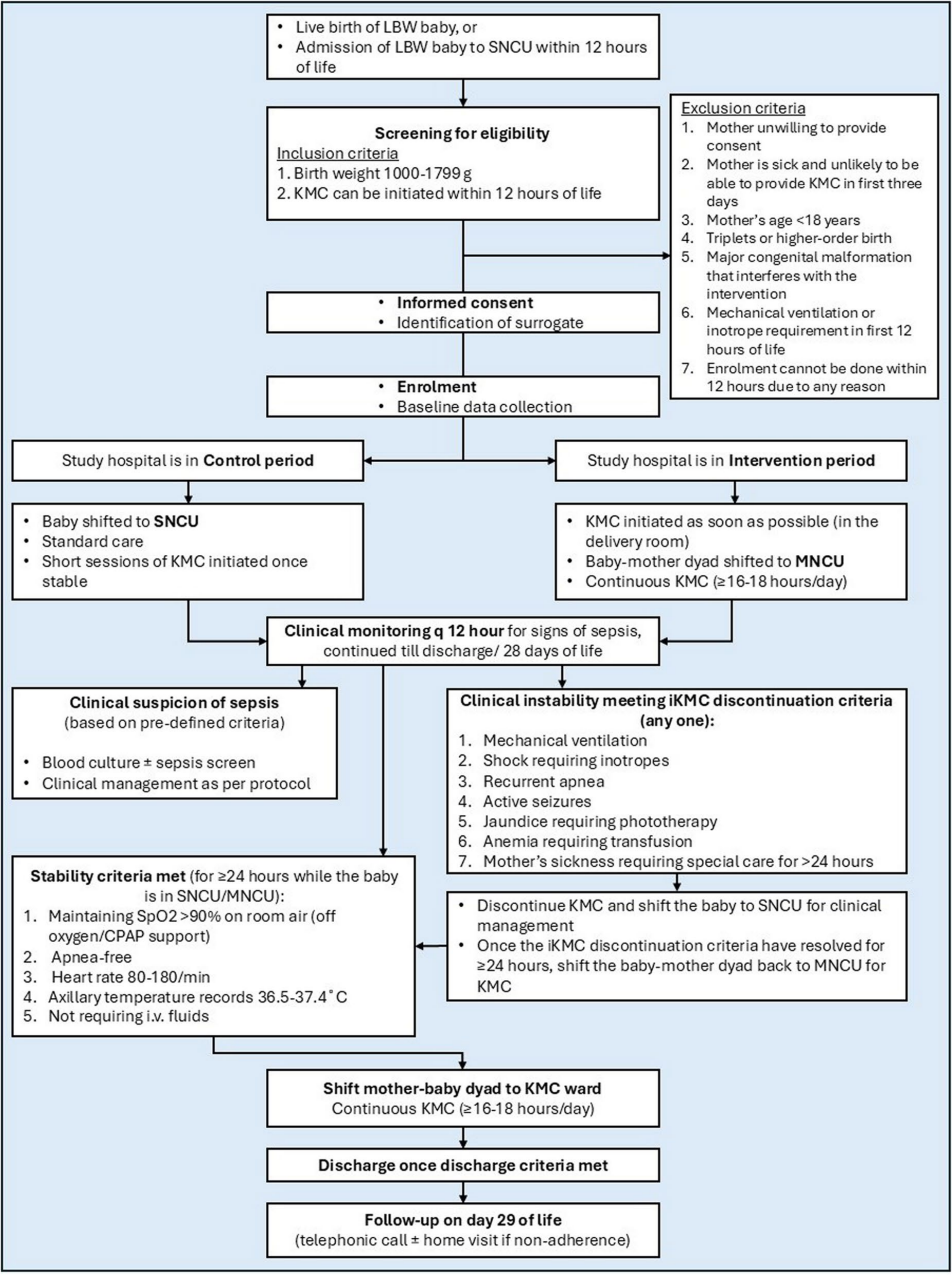
Overview of the patient flow. Abbreviations: LBW low birth weight, SNCU special newborn care unit, KMC kangaroo mother care, MNCU mother newborn care unit, iKMC immediate kangaroo mother care, CPAP continuous positive airway pressure

Figure 4 depicts an overview of the patient flow in the trial.

### Data collection and management

The outcome assessment research nurses will evaluate outcomes during both intervention and control periods using the same methods and procedures. Throughout the hospital stay, outcomes will be assessed by conducting neonatal evaluations every 12 h in the SNCU and MNCU wards, observing care provided, and reviewing medical records (including notes and treatment/feeding charts). Steps will be taken to ensure participant retention and complete follow-up. The outcome assessment nurse will make a phone call on day 29 of age to determine outcomes at the end of the neonatal period. If there is no contact for 3 days by phone, a home visit will be made at the address provided during enrollment.

All the neonatal and maternal data will be entered into the standardized case record forms by the research nurses and subsequently entered into an electronic data capture platform (Open Data Kit platform, 2025 Get ODK Inc.). The database application access will be provided to the staff through a laptop/desktop as determined by the project administrator. The site research officers will ensure the timely completion of the data and conduct random checks on the forms for validation purposes. A secure login password will be used for data extraction, and the access rights to delete it will remain with the principal investigator.

### Statistical analysis

The study flow will be depicted as per the flowchart suggested in the CONSORT statement—extension for stepped wedge cluster randomized trials [15]. Data will be analyzed using Stata 15.1 (StataCorp, USA) and the R Statistical Software. The analysis will be one-sided with a significance level of 0.025 and will be done as per intention-to-treat (ITT) principle.

Baseline characteristics will be summarized as proportions, mean and SDs, or median and IQR. For the primary composite outcome of all-cause mortality or culture-positive sepsis, relative risk with 95% confidence intervals will be reported. A generalized linear mixed model with a log link function will be used to analyse the effect of iKMC on all-cause mortality or culture-positive sepsis, and relative risk with 95% confidence intervals will be reported. The primary analysis will be carried out after adjusting for time and cluster effect as potential confounders due to imbalance of the study design (stepped wedge cluster randomized design) with respect to time. As a sensitivity analysis, the primary analysis model will be re-run with the additional covariates of sex, gestational age, and birth weight included.

To account for the possible lag in the uptake of intervention, we shall assume the 14 days as the “transition period,” and observations during this lag period will be considered under the “intervention period.” The following hypothesis will be tested:

1 $$H_0:\;RR\geq1\;vs\;H_A\;:RR\;<1$$

where RR is the relative risk of all-cause mortality or culture-positive sepsis under iKMC compared to control.

The primary analysis will be performed using an intention-to-treat (ITT) analysis population, defined as all eligible and enrolled neonates. As a sensitivity analysis, the primary analysis will also be conducted on the site ITT population, defined as all neonates of birth weight 1–1.799 kg, *regardless of eligibility and enrollment*, born at the study sites during the study period. This analysis will be done using de-identified aggregate-level data from the study sites, in view of ethical concerns with the inclusion of individual-level data of non-enrolled neonates. This analysis will not be adjusted for covariates.

Subgroup analyses are planned to explore outcomes differences by birth weight (1000–1499 g or 1500–1799 g), birth weight category (small-for-gestational age (SGA) or non-SGA), time of initiation of iKMC (< 2 h or 2–11 h), and average daily duration of skin-to-skin contact (< 8 h, 8 to < 16 h, or ≥ 16 h).

Secondary binary endpoints will be analyzed with the same analysis method used for the primary endpoint. The number of hospital-free days will be analyzed as an ordinal outcome, with death considered as the worst possible measure, i.e., −1. An analysis approach similar to that used for the primary endpoint will be employed, incorporating a logit link function to account for the ordinal nature of the outcome, while adjusting for clustering and time effects inherent in the stepped wedge design. For time-to-event endpoints (time to neonatal mortality and time to culture-positive sepsis), no formal statistical tests will be performed. However, log-rank tests will be reported for exploratory purposes along with the Kaplan–Meier curves.

Details of the statistical models and analyses have been provided in the Statistical Analysis Plan (SAP).

### Data privacy

With appropriate technical measures, personal information will be securely recorded to ensure adequate protection, including safeguards against unauthorized or illegal handling and accidental loss. Patient privacy will be upheld by removing all identifying details, which will subsequently be encrypted with scientific codes. The data will be stored throughout the study and retained for as long as necessary. Once the intended objectives are met, personal information will be deleted and no longer kept.

### Consent

During the control period, healthcare providers will continue with the recommendations of the national/WHO guidelines on KMC. Even during the intervention period, the study will not introduce any “new” intervention but will implement the existing WHO recommendations in all participating units. However, due to logistic challenges, the implementation will be done sequentially. Thus, the intervention unit will be the SNCUs and not the participants. The relevant clearances have been obtained from the state-level authorities.

We will take both cluster-level and individual-level consent. Consent shall be obtained from the hospital administration and doctors for participation in the study. The Chief Medical Officer (CMO) will be the co-PI and supervise the implementation of the study in the given district. In addition, we will seek written informed consent from individual participants (mothers or any legally authorized representative). Further, consenting mothers will be asked to identify one or two adult women relatives or friends of their choice who could act as their surrogates for providing SSC when and if they are not able to do so. Surrogates will have their role in the study explained to them if the mother-infant pair is randomly assigned to the intervention group.

### Training and standardization

The healthcare staff in the SNCUs are trained to deliver neonatal care using the basic care package for newborns and mothers. All relevant staff in the neonatal unit are trained to support KMC for very small infants. This includes the proper technique for safely wrapping and positioning the neonate’s head during KMC, especially when the neonate is asleep.

As outlined in the WHO manual for small infants, the standard of care includes monitoring, maintaining body temperature, supporting breastfeeding, and ensuring hygiene for all infants. It also involves providing access to intravenous fluids, antibiotics, and respiratory support with safe oxygen therapy and bubble CPAP if needed. To enhance the implementation of WHO Essential Care for Small Babies, all participating SNCUs will receive support.

All research team members involved in the study will receive thorough initial training for 5 to 7 days before site initiation. Further, during implementation, their activities will be overseen by competent research officers who will ensure adherence to the SOPs. Research nurses will also receive training in screening and enrolling participants, providing KMC support, and measuring outcomes. The teams will also undergo regular training using a standardized study operations manual. Furthermore, all research staff will receive training in building rapport and communicating effectively with mothers and families.

The monitoring team and site Principal Investigators (PIs) will conduct internal checks to maintain the study’s quality. These checks will consist of supervised observations and random independent assessments. Supervised observations will involve weekly supervision of the activities implemented by the monitoring team/PI. Independent checks will involve 5% of observations reviewed independently by the nodal team.

### Monitoring and quality assurance

We will implement various quality assurance measures to ensure the data’s accuracy, thoroughness, and timeliness. The Clinical Development Services Agency (CDSA) in Faridabad, Haryana, India will act as study monitors, overseeing the quality of procedures, data collection, and documentation. The following quality assurance steps will be followed:

- We will collaborate with investigators from all sites, subject experts, and district authorities to develop a shared study protocol, implementation framework, and data collection tools.
- The research team, led by investigators and the project coordinator, will ensure adherence to the study protocol and uphold rigorous implementation. A monitoring team will conduct regular oversight through field visits (every 3 months), with members from the nodal center participating.
- Study sites will establish and follow internal processes for quality checks, including ensuring data consistency, completeness, and accuracy across various data components.
- The electronic data capture system/platform for quantitative data will have checks such as range/limit validations, logical sequences, and skips to minimize errors and facilitate timely submission/transmission. Qualitative data will be collected by trained team members and analyzed by experienced individuals.

Research officers responsible for their respective areas will manage and review the database. The data entry system will include mechanisms for automatically performing range and consistency checks.

### Trial oversight

An independent data safety and monitoring board (DSMB) will be constituted. The DSMB will include at least five members with expertise in clinical trials, statistics, newborn care, and ethics. The DSMB will safeguard the interests of trial participants, potential participants, investigators, and sponsors. They will also oversee the overall execution and quality of the trial to maintain its integrity and reliability. Further, they will evaluate the safety and initial effectiveness of the trial’s intervention based on predetermined data assessment time points and make recommendations regarding continuation or early termination due to indications of benefit/harm or futility. The Technical Advisory Group (TAG) for the trial will consist of neonatologists, infectious disease experts, and public health experts of national and international reputation.

### Interim analysis

Two interim analyses will be performed once the primary and secondary outcomes after 4th and 7th sites have been randomized and have completed their 3-month intervention period. The purpose of these interims is to assess the treatment effect as data accumulates. The O’Brien-Fleming rule will be used to stop the trial for early success. An independent statistician will perform the interim analysis. The statistician will report to the DSMB. The DSMB will have unblinded access to all data and will discuss the interim analysis results and recommendations regarding trial continuation with the investigators in a joint meeting. The investigators will decide on the modification/continuation of the trial and will report to the institute ethics committee (IEC).

## Discussion

A significant fraction of the 2.5 million annual global neonatal deaths is preventable [16]. An estimated 58% of prematurity-related mortality and 84% of infection-related mortality can be prevented with evidence-based interventions [17]. However, efforts aimed at reducing newborn mortality are hampered significantly due to stark social, geographic, and economic disparities, exemplified aptly by the state of neonatal health in Sub-Saharan Africa and southern Asia. The need of the hour is to find contextual solutions that are potent, feasible, and sufficiently cost-effective. We aim to evaluate the impact of one such intervention—iKMC—on neonatal mortality and sepsis in low-birth-weight neonates in district hospitals in Chhattisgarh, India.

The study sites were chosen to effectively understand the impact and feasibility of implementing iKMC in community settings within LMICs. Chhattisgarh, a state in central India, has a significant tribal population residing in hard-to-reach areas. The state’s NMR is 32.4 per 1,000 live births, compared to the national NMR of 24.9 and a global NMR of 17 [18]. Approximately 70% of deliveries in Chhattisgarh occur in public health facilities, including SNCUs, which provide secondary-level care to small and sick neonates. More than half of all neonatal deaths in SNCUs in Chhattisgarh are associated with low birth weight (LBW) and prematurity, with LBW increasing the likelihood of mortality threefold and very low birth weight status 11-fold [19]. Similarly, there is a considerable burden of neonatal sepsis and its related mortality [4]. iKMC has the potential to be a highly cost-effective intervention for reducing neonatal mortality and sepsis in this area.

The WHO has strongly recommended immediate KMC for preterm or LBW neonates [20]. However, compliance with immediate KMC remains low in most parts of the world due to several reasons: (a) iKMC requires an initial investment to establish mother-newborn intensive care units (M-NICU) and train healthcare professionals, (b) safety concerns arise when initiating iKMC before stabilizing the neonate, particularly in resource-constrained settings with limited monitoring capabilities, (c) the evidence for its benefits is unclear, with the OMWANA study not indicating any mortality benefit, although it shows promising economic benefits [21]. Given the favorable benefit-to-harm ratio overall and the anticipated logistical challenges in implementation, we structured the study as a pragmatic trial utilizing a stepped-wedge cluster design.

We anticipate some challenges during the trial implementation. Although we selected the study sites based on the availability of MNCU or adequate space for MNCU, significant efforts will be necessary to functionalize the MNCU at each site. We will deploy trained research staff round the clock at each study site to ensure a timely participant enrollment process, including screening and informed consent. Another group of research nurses will be assigned to ensure and record adherence to KMC, aiming for over 16 h per day, while monitoring for signs and symptoms of sepsis. Since iKMC is not routinely practiced at the study sites, we expect some initial apprehension about its implementation. A transition period of 14 days has been established as a safeguard until iKMC is optimally implemented during the intervention period at each site. Despite these measures, we anticipate some non-adherence to the intervention.

In addition to clinical outcomes, we also aim to explore the pathophysiological mechanisms underlying the effect of iKMC on neonatal sepsis. iKMC is believed to influence the microbial pathogen profile, leading to a predominance of Gram-positive bacteria [11]. We will examine culture and antimicrobial resistance pattern data for all cases of culture-positive sepsis. Additionally, we will gather skin and stool swab samples from a subset of enrolled neonates on day 3 and day 7 to illuminate the changes in microbial colonization in neonates exposed to early KMC.

The successful implementation and positive outcomes of iKMC will alleviate clinicians’ safety concerns regarding preterm LBW neonates, particularly during the first few days of life. It will also encourage policymakers to enhance infrastructure (MNCUs) and human resource capacity for implementing iKMC for LBW neonates, stimulating a paradigm shift in neonatal care. To contextualize the potential benefits, an intervention that leads to a 10% reduction in neonatal mortality among low-birth-weight neonates can prevent approximately 200,000 neonatal deaths each year. This intervention could represent a significant step toward achieving the goal of a single-digit neonatal mortality rate in all regions by 2035.

## Supplementary Information

Supplementary Material 1: Supplementary Table 1: Definitions of sepsis-related terminology.

Supplementary Material 2: PRISM study Statistical Analysis Plan.

Supplementary Material 3: SPIRIT checklist.

## Data Availability

At the completion of the study, deidentified data that support the findings of this study will be made openly available on a public repository, after getting necessary approval from the Department of Health and Family Welfare, Chhattisgarh, India. We will disseminate the findings of the study through formal meetings with stakeholders and international conferences. We plan to publish the findings in peer-reviewed journals in an open-access format. As required by our sponsor’s open access policy, all publications shall be under the Creative Commons Attribution 4.0 Generic License (CC BY 4.0). Further, we shall ensure that the data underlying published research results will be accessible and open to the public.

## Abbreviations

LBW: Low-birth weight
LMIC: Low- and middle-income countries
KMC: Kangaroo mother care
iKMC: Immediate kangaroo mother care
WHO: World Health Organization
RR: Relative risk
CI: Confidence interval
PRISM: Protective Role of Immediate KMC in neonatal Sepsis and Mortality
SPIRIT: Standard Protocol Items: Recommendations for Interventional Trials
SNCU: Special newborn care unit
CPAP: Continuous positive airway pressure
MNCU: Mother newborn care unit
AIIMS: All India Institute of Medical Sciences
RN: Research nurse
SSC: Skin-to-skin contact
CLSI: Clinical and Laboratory Standards Institute
SD: Standard deviation
IQR: Interquartile range
ITT: Intention-to-treat
SGA: Small-for-gestational age
CMO: Chief Medical Officer
SOP: Standard operating procedures
PI: Principal investigator
CDSA: Clinical Development Services Agency
DSMB: Data safety and monitoring board
TAG: Technical advisory group
IEC: Institute ethics committee
NMR: Neonatal mortality rate
MNICU: Mother newborn intensive care unit
