# Supplementary material for: Effect of immediate kangaroo mother care (iKMC) on neonatal mortality and culture-positive sepsis in low-birth-weight neonates in district hospitals in Chhattisgarh, India (PRISM study): protocol for a stepped-wedge cluster randomized trial
Source: Trials. 2025 Oct 9;26:397. doi: 10.1186/s13063-025-09083-3 (PMC12512956; doi:10.1186/s13063-025-09083-3)
Supplement: Supplementary file 1 — Supplementary Material 1: Supplementary Table 1: Definitions of sepsis-related terminology. [file 13063_2025_9083_MOESM1_ESM.pdf]

Supplementary Table 1: Definitions of sepsis-related terminology

Diagnosis	Working definition
Suspected sepsis	Age of the neonate <28 days<br>AND<br>Presence of ANY ONE of the risk factors/ clinical symptoms/signs from the following list, for which the neonate has no other known or reliable explanation:<br>Perinatal risk factors<br>1. Foul-smelling liquor<br>2. At least two of the following: (i) spontaneous prematurity and preterm pre-labor rupture of membranes (PPROM; irrespective of duration of rupture of membranes) (ii) Febrile illness in the mother with suspected bacterial infection warranting start of antibiotics by attending clinician in the week prior to delivery (iii) rupture of membranes >24 hours (iv) unclean vaginal examination(s) during labor<br><br>Clinical symptoms/signs<br>1. No movement or movement only when stimulated<br>2. Refusal to feed<br>3. Severe chest in-drawing or increased oxygen requirement or need for respiratory support<br>4. Grunting<br>5. New onset apnea or increased severity or frequency of apnea in a baby who already is having apneas<br>6. Cyanosis or desaturation needing oxygen therapy or respiratory support (or increase in oxygen requirement/deterioration in a baby who is already receiving oxygen therapy)<br>7. Fever or hypothermia (>37.5˚C or <36.5˚C)<br>8. Tachycardia or episodes of bradycardia (>180/min or <100/min)<br>9. Capillary refill time (CRT) > 3 sec<br>10. Mottled skin or other evidence of shock<br>11. Erythema in the skin around umbilical stump (extending to >1 cm)<br>12. Lethargy or drowsiness<br>13. Convulsions<br>14. Abnormal posturing<br>15. Hypotonia or floppiness<br>16. Bulging fontanelle<br>17. Vomiting or abdominal distension<br>18. Bleeding<br>19. Sclerema
Culture-positive sepsis	Neonate with ‘suspected sepsis’ (see above) AND isolation of a recognized pathogen from blood, cerebrospinal fluid, or other sterile body fluids.
Clinical sepsis	Neonate with ‘suspected sepsis’ AND negative blood culture (or blood culture deemed to have grown a commensal) AND one or more of the following:<br>1. Clinical course consistent with sepsis and there was no alternative explanation for the clinical symptom(s)<br>2. Positive laboratory criteria (at least one of the following: white blood cells count <4.0 x 10^9 cells/L; absolute neutrophil count < 1.5 X 10^9 cells/L; IT ratio>0.2; C-reactive protein (CRP) > 6 mg/L)
Early-onset sepsis	Occurrence (i.e. time of onset of symptoms) of culture-positive or clinical sepsis at or before 72 h of life
Late-onset sepsis	Occurrence (i.e. time of onset of symptoms) of culture-positive or clinical sepsis after 72 h of life
