# Supplementary material for: Effect of immediate kangaroo mother care (iKMC) on neonatal mortality and culture-positive sepsis in low-birth-weight neonates in district hospitals in Chhattisgarh, India (PRISM study): protocol for a stepped-wedge cluster randomized trial
Source: Trials. 2025 Oct 9;26:397. doi: 10.1186/s13063-025-09083-3 (PMC12512956; doi:10.1186/s13063-025-09083-3)
Supplement: Supplementary file 2 — Supplementary Material 2: PRISM study Statistical Analysis Plan. [file 13063_2025_9083_MOESM2_ESM.pdf]

Effect of immediate kangaroo mother care (iKMC) on neonatal mortality and culture-positive sepsis in low-birth-weight neonates in district hospitals in Chhattisgarh, India (PRISM study): protocol for a stepped-wedge cluster randomized trial

(PRISM: **P**rotective **R**ole of **I**mmediate KMC in Neonatal **S**epsis and **M**ortality)

Statistical Analysis Plan

Version 1.0, dated 19 June 2025

Summary of SAP revisions

**Version number**	**Revision Date**	**Reason for revision**	**Revision approved by**
Version 1.0			

Table of Contents

Contents	Page number
1. Background	3
2. Research Question and Objectives	4
3. Methodology	5
4. Outcomes	7
5. Study Population	11
6. Protocol Adherence	12
7. Analysis Populations	12
8. Analysis	12
9. Interim Analysis and Trial Stopping Guidance	15
10. Safety Monitoring	16

1. Background

Kangaroo mother care (KMC), i.e., continuous skin-to-skin contact of preterm low birth weight (LBW) neonates with their mothers' chest and feeding exclusively with breast milk, is one of the most effective interventions for preventing neonatal mortality. Traditionally, KMC is initiated in LBW infants after stabilization, which delays the initiation of KMC in most preterm neonates: the median age of initiation of KMC varied from 3 to 24 days in the studies on KMC. Given that about 45% of neonatal deaths occur within 24 hours after birth and 80% during the first week of life, most deaths in LBW neonates occur before KMC can be initiated.

The effect of initiating kangaroo mother care immediately after birth—immediate kangaroo mother care (iKMC)—has recently been evaluated in a multi-country randomized trial in five level-3 hospitals in Ghana, India, Malawi, Nigeria, and Tanzania (funded by BMGF and sponsored by WHO). The study showed a significant reduction in the risk of neonatal mortality (by day 28 of life) in the iKMC group—12.0% vs. 15.7%; RR 0.75; 95% CI 0.64 to 0.89. However, another RCT conducted in five hospitals in Uganda (OMWaNA trial) among neonates weighing 700–2000 g without life-threatening clinical instability showed that KMC initiated before stabilization did not reduce early neonatal mortality, i.e., mortality within seven days of age. Thus, the two trials provide conflicting evidence on the effect of KMC initiated before the clinical stabilization of neonates on reducing neonatal mortality rates.

A post-hoc analysis of the WHO iKMC trial demonstrated a significant reduction in the incidence of suspected sepsis in the intervention group – 22.9% vs. 27.8%; adjusted RR 0.82; 95% CI 0.73 to 0.93. The risk of sepsis-related mortality was also significantly lower (4.4% vs. 6.9%; adjusted RR 0.63; 95% CI 0.47 to 0.85. However, there was no reduction in the incidence of culture-positive sepsis in the iKMC group – 25/1609 (1.6%) vs. 30/1602 (1.9%); RR 0.83; 95% CI 0.49 to 1.40, probably due to the low baseline incidence and consequently low power. The OMWaNA trial from Uganda reported no difference in the incidence of 'suspected or confirmed neonatal sepsis' between the KMC and control groups.

Given the evidence or its lack thereof on the beneficial effects of iKMC on neonatal mortality and culture-positive sepsis, it is imperative to evaluate the impact of immediate KMC in preterm LBW neonates admitted to health facilities in low- and middle-income countries.

2. Research Question and Objectives

2.1 Research question

Among low birth weight neonates (1000-1799 g at birth) admitted within 12 hours of life to the special care newborn units (SNCU) of district hospitals (P), does immediate kangaroo mother care (iKMC) initiated within 12 hours of life (I) reduce the incidence of all-cause neonatal mortality or culture-positive sepsis (O) compared to conventional care (C), i.e., care in a radiant warmer until their condition stabilizes, followed by the initiation of KMC?

2.2 Objectives

Primary

To evaluate the effect of immediate kangaroo mother care (iKMC) on the incidence of all-cause neonatal mortality and/or culture-positive sepsis in neonates with a birth weight of 1000-1799 g who are admitted to the special newborn care units of district hospitals in Chhattisgarh, India.

Secondary:

To evaluate the effect of immediate kangaroo mother care (iKMC) on:

a. All-cause neonatal mortality
b. Culture-positive sepsis in the first 28 days of life
c. Time to neonatal mortality
d. Early (<7 days of life) neonatal mortality
e. Incidence of early-onset (<72 hours of life) and late-onset (≥72 hours of life) culture-positive sepsis
f. Incidence of clinical sepsis
g. Exclusive expressed breast milk feeding or exclusive direct breastfeeding at discharge
h. Hospital-free days
i. Pathogen profile and antimicrobial resistance pattern in neonates with culture-positive sepsis
j. Skin and gut colonization of the neonates at day 3 (± 24 hrs) and day 7 (± 24 hrs)

3. Methodology

This analysis plan is based on the current version of the protocol, Version 1.0, dated June 14, 2025.

3.1 Study design*:* Stepped-wedge cluster randomized controlled trial

3.2 Setting*:* Special newborn care unit (SNCU) of 10 district hospitals in Chhattisgarh, India.

3.3 Study duration: 42 months

3.4 Inclusion criteria:

All live low birth weight (1000-1799 g) neonates admitted to the study hospitals, irrespective of place of birth, in whom KMC can be initiated within 12 hours of life.

3.5 Exclusion criteria:

1. Neonates born to mothers who are either unwilling to give consent or are ill and unlikely to be able to provide KMC within the first three days after birth.
2. Neonates born to mothers younger than 18 years old.
3. Triplets or higher-order births.
4. Major congenital malformation that interferes with the intervention, or the intervention interferes with the required care for the congenital malformation.
5. Neonates requiring intubation and mechanical ventilation or inotropes for shock within the first 12 hours of life.
6. If the mother-infant pair cannot be enrolled within 12 hours of the neonate's birth for any reason (such as if the mother is admitted elsewhere/deceased)

3.6 Randomization of clusters

In a stepped-wedge study, the intervention is gradually implemented in steps at the participating clinical sites. The order of implementing the intervention will be determined using a computer-generated random sequence placed in sequentially numbered, opaque sealed envelopes at the coordinating centre. We will have a baseline period of six months before randomizing the first site. The subsequent study period will be divided into ten phases, each lasting three months. Before each step, the envelope will be opened, and the clinical site randomly selected for that phase will be notified about initiating iKMC and ensuring care for the neonate in the mother-newborn care unit (MNCU). A five- to seven-day training session will be conducted for the research staff prior to rolling out the intervention. Therefore, the clinical sites will be informed about the initiation of the intervention at their site 14 days before the scheduled phase. These 14 days, starting after three

months of the intervention period completed at the previous site, will be referred to as the 'transition period.' The intervention period will begin once the transition period is over and will continue for three months before the next site is randomly selected for the intervention. All ten clinical sites will have received the intervention by the end of the ten steps. Thus, each site will have its pre-intervention period as the control period (Figure 1).

Figure 1: The stepped-wedge design for the study

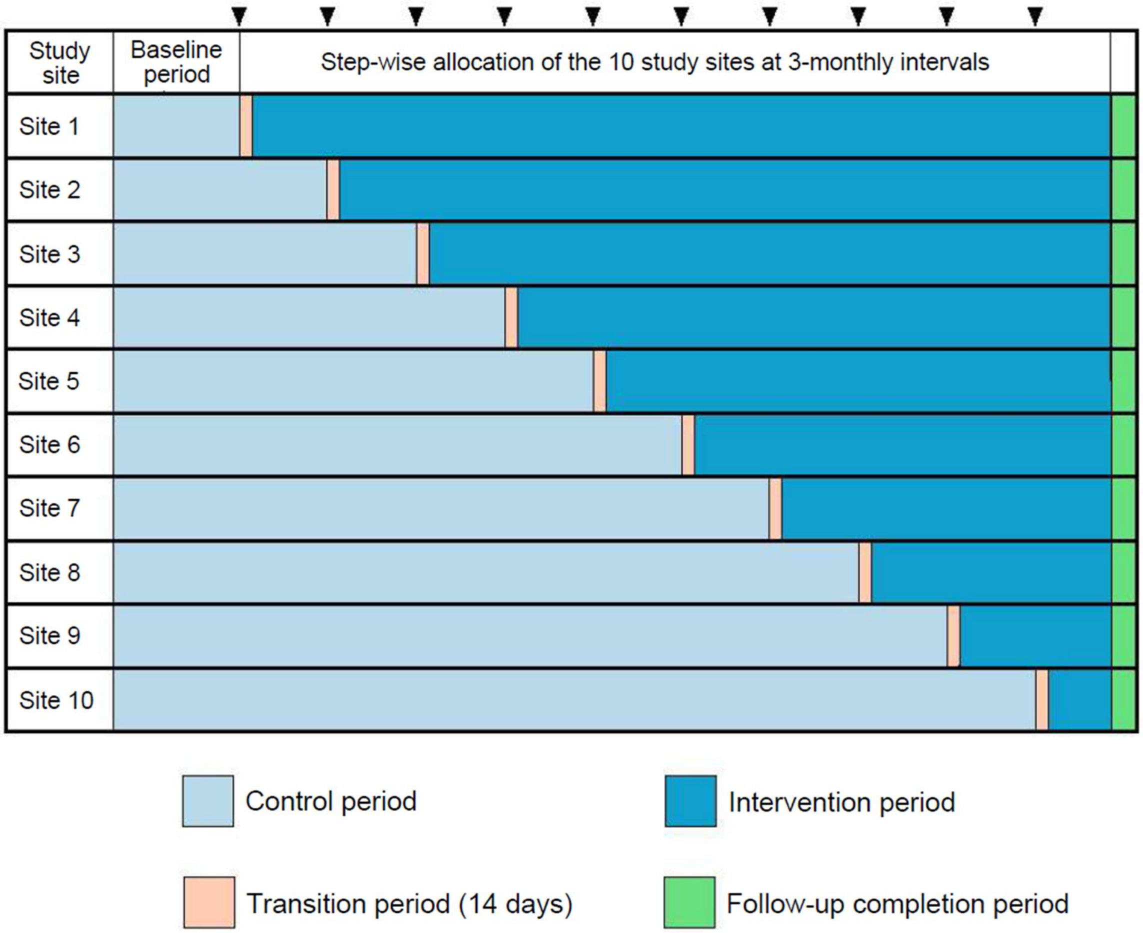

3.7 Sample size calculation

Based on preliminary data collected from the study sites, we found a 27% incidence of mortality or culture-positive sepsis in neonates with birth weights of 1-1.799 kg. Assuming a 22% relative reduction in its incidence in the intervention arm (i.e., 21.1% mortality or culture-positive sepsis), variable cluster sizes cantered around a mean rate of 36 neonates per 3-month phase, an intra-cluster correlation of 0.01, ten phases preceded by a baseline period of 6 months, 14-days

transition periods, and a one-sided alpha of 0.025, we calculated a power of 92% with an average total sample size of 4920. No adjustments were made for cluster attrition as the risk of attrition was considered low.

Following the baseline period, we plan to review control incidence rates to re-evaluate the sample size and study duration. Additionally, the final sample size may be lower if the adaptive trial is stopped early due to significant benefits or futility considerations.

4. Outcomes

4.1 Study objectives and endpoints

Table 1 depicts the study objectives and corresponding endpoints.

Table 1: Study objectives and corresponding endpoints

Objective	***Endpoint***
**Primary**	
To evaluate the effect of immediate kangaroo mother care (iKMC) on the incidence of all-cause neonatal mortality and/or culture-positive sepsis in neonates with birth weights of 1000-1799 g and admitted to level 2 units of the district hospitals	• All-cause neonatal mortality and/or culture-positive sepsis until 28 days of life
**Secondary**	
To evaluate the effect of immediate kangaroo mother care (iKMC) on the incidence of all-cause neonatal mortality in neonates with birth weights of 1000-1799 g and admitted to level 2 units of the district hospitals	• All-cause neonatal mortality until 28 days of life
To evaluate the effect of immediate kangaroo mother care (iKMC) on the incidence of culture-positive sepsis in neonates with birth weights of 1000-1799 g and admitted to level 2 units of the district hospitals	• Culture-positive sepsis until 28 days of life
To evaluate the effect of iKMC on time to neonatal mortality	• Time to all-cause neonatal mortality, using Kaplan-Meier survival estimates
To evaluate the effect of iKMC on early neonatal mortality	• All-cause neonatal mortality within first 7 days of life
To evaluate the effect of iKMC on the incidence of early-onset and late-onset culture-positive sepsis	• Early-onset (<72 hours of life) culture-positive sepsis<br>• Late onset (≥72 hours of life) culture-positive sepsis<br>• Time to culture-positive sepsis, using Kaplan-Meier survival estimates
To evaluate the effect of iKMC on incidence of clinical sepsis	• Clinical sepsis in neonates within 28 days of life
To evaluate the effect of iKMC on exclusive expressed breast milk feeding or exclusive direct breastfeeding at discharge	• Exclusive expressed breast milk feeding or exclusive direct breastfeeding at discharge
To evaluate the effect of iKMC on the number of hospital-free days	• Number of hospital-free days, defined as the number of days, up to 28 days of life, not admitted to the hospital

**Exploratory**	
To describe the pathogen profile and antimicrobial resistance pattern in neonates with culture-positive sepsis	• Number organisms isolated from neonates<br>• Organisms with resistance to various antimicrobial agents
To describe the skin and gut colonization of the neonates at day 3 (± 24 hrs) and day 7 (± 24 hrs) in the two periods	• Alpha and beta diversity of the organisms colonizing skin and gut at day 3<br>• Alpha and beta diversity of the organisms colonizing skin and gut at day 7

4.2 Schedule of outcome assessment

Figure 2 shows the schedule of outcome assessment.

Figure 2: Trial schedule of enrolment, interventions, and assessments

	**Pre-enrolment**	**Enrolment**	**Post-allocation**					**Close-out**	**Laboratory confirmation**
**Timepoint**	Birth/hospital admission	Within 12 hours	Stay in MNCU/ SNCU/ KMC ward				After hospital discharge until day 29	Day 29	At nodal centre
			Every 12 hours till discharge	Day 3	Day 7	At discharge/ day 28			
**Enrolment**									
Eligibility screening	X								
Informed consent	X								
Allocation	As per study site allocation								
**Intervention**									
iKMC		X	X	X	X	X			
Routine KMC (control)			Once stable	X	X	X			
Standard preterm care	X	X	X	X	X	X			
**Assessment**									
Daily duration of SSC			X	X	X				
Sepsis suspicion			X	X	X	X			
Final labelling of sepsis						X			
Neonatal mortality			X	X	X	X	X	X	
Early neonatal mortality				X	X				
Exclusive expressed breastmilk feeding						X			
Duration of hospital stay						X			
Skin and gut colonization				X	X				X

Abbreviations: MNCU mother newborn care unit, SNCU special newborn care unit, KMC kangaroo mother care, SSC skin-to-skin contact

4.3 Timing of final analysis

All outcomes will be analyzed collectively after the final participant has completed follow-up and data has been cleaned.

5. Study population

5.1 Trial flow chart

The study flow will be depicted as per the flowchart suggested in the CONSORT statement-extension for stepped wedge cluster randomized trials.

5.2 Screening data

The total number of low-birth-weight neonates admitted to the study sites during the study period will be described.

5.3 Eligibility

The number of participants screened for eligibility and the outcome of screening will be presented, including the reasons for ineligibility.

5.4 Withdrawal/ follow-up

The number and proportion of mother-newborn dyads who temporarily or permanently withdraw from the intervention will be reported, along with reasons for withdrawals. For temporary withdrawals, the number and proportion of those restarting the intervention at a later stage will be described. The number and proportion of the participants who are followed up for the primary outcome at death/ on day 29 will be described, with further description of the following:

A. Died/ alive
B. Culture-positive sepsis/ death/ both/ none
C. Followed-up telephonically/ home visit

5.5 Baseline characteristics

Baseline characteristics will be reported in a tabular form for the intention-to-treat dataset. Baseline characteristics will be summarized as proportions, mean and SDs, or median and IQR. No significance tests will be performed to test for differences at baseline.

6. Protocol adherence

Adherence for the intervention will be monitored with the daily amount of time spent in skin-to-skin contact. Adherence will be defined as:

a. For intervention (iKMC), skin-to-skin contact should be initiated within 12 hours of life and performed at the desired daily duration of 16 hours/day.
b. For control (standard KMC), skin-to-skin contact should be initiated once stability criteria are met. In SNCU, one or more short sessions of skin-to-skin contact will be done. Once mother-baby dyad gets shifted to the KMC ward, a desired daily skin-to-skin contact duration of 16 hours/day will be targeted.

7. Analysis populations

7.1 Participant Intention-to-Treat (ITT)

The participant intention-to-treat (ITT) analysis population includes all eligible and enrolled neonates within each site, analysed based on their assigned intervention during the designated time period, regardless of whether they actually received the intervention. *Note, the ITT principle here applies to the neonate and all eligible and enrolled neonates at the site are included in the primary outcome.*

7.2 Site Intention-to-Treat (ITT)

The site intention-to-treat (ITT) analysis population includes all neonates of birth weight 1-1.799 kg, regardless of eligibility and enrolment, born at the study sites during the study period. *Note, the ITT principle here applies to the site and all neonates (of birth weight 1-1.799 kg), regardless of eligibility and whether they received the intervention, at the site are included in the primary outcome.*

8. Analysis

Data will be analysed using Stata 15.1 (StataCorp, USA) and R (R program for statistical computing). The analysis will be one-sided with a significance level of 0.025 and will be done as per intention-to-treat principle.

8.1 Primary analysis

A generalized linear mixed model with a log link function will be used to analyse the effect of iKMC on all-cause mortality or culture-positive sepsis and relative risk with 95% confidence intervals will be reported. Let Y_i denote the primary endpoint, i.e., the indicator for the event of death or culture-positive sepsis for patient i. Moreover, let γ_{ijt} denote the probability of an event for patient i, enrolled in cluster (site) j during time period t. The primary analysis model formulation is:

$$\log(\gamma_{ijt}) = \mu + \beta_t T + \theta X_{jt} + u_j + \boldsymbol{\gamma} \boldsymbol{z_i}$$

where the intercept μ is the log-transformed baseline control rate, β_t is the time trend parameter for time period t, u_j is the random effect associated with cluster j where $u_j \sim N(0, \tau^2)$, X_{jt} is the indicator denoting if cluster j at time t is randomized to control ($X_{jt} = 0$) or treatment ($X_{jt} = 1$), and θ is the treatment effect on the log scale. The time effect parameters, β, refer to the fixed effects representing each step (e.g., a 3-month interval). This accounts for systematic differences between steps, such as a learning effect as the intervention is implemented. The variables $\boldsymbol{z_i} = (z_{i1}, \dots, z_{ip})$ represent any baseline covariate included in the model.

To account for the possible lag in the uptake of intervention, we shall define the 14 days as the 'transition period,' and observations during this lag period will be considered under the 'intervention period'. The following hypothesis will be tested:

H_0: RR ≥ 1 vs H_A: RR < 1

where RR is the relative risk of all-cause mortality or culture-positive sepsis under iKMC compared to control. This corresponds to testing that $\theta \geq 0$ and is evaluated using a one-sided alpha level of 0.025.

The primary analysis will be performed using the participant intention-to-treat (ITT) analysis population, defined as all eligible and enrolled neonates. As sensitivity analysis, the primary analysis will also be conducted on the site ITT population, defined as all neonates of birth weight 1-1.799 kg, regardless of eligibility and enrolment, born at the study sites during the study period. This analysis will be done using de-identified aggregate-level data from the study sites, in view of ethical concerns with inclusion of individual-level data of non-enrolled neonates. This analysis will not be adjusted for covariates. Additionally, the primary analysis will be repeated considering the 'transition period' in the 'control period'.

8.1.1 Adjustments for potential covariates

The primary analysis will be carried out after adjusting for time and cluster effect as potential confounders due to imbalance of the study design (stepped wedge cluster randomized design) with respect to time. As a sensitivity analysis, the primary analysis model will be re-run with the additional covariates of sex, gestational age, and birth weight included.

8.1.2 Subgroup analyses

Subgroup analyses are planned to explore outcomes based on:

a. Birth weight (< 1000-1499 g or 1500-1799 g)
b. Birth weight category (small-for-gestational age (SGA) or non-SGA)
c. Time of initiation of iKMC (<2 hours or 2-11 hours), and
d. Average daily duration of skin-to-skin contact (<8 hours, 8 to <16 hours, or ≥16 hours).

All subgroup analyses will be performed on the participant ITT population.

8.1.3 Dropouts and missing data

The data management team will be responsible for reviewing all data for completeness and correctness. They will contact the study site team regarding any missing data and other queries. Missing data will be sought until received or confirmed as not available. A trial database will be maintained on ODK database (Open Data Kit platform, 2025 Get ODK Inc.) and data monitoring, including validation, verification, and cleaning.

Participants with missing data on the primary outcome will be excluded from the primary analysis. Every effort will be made to minimize the missing data, and the expected proportion of missing data is small. Sensitivity analyses will be done if the missing data for the primary outcome is > 5% including best- and worst-case scenario analyses. Additionally, a tipping point analysis will be performed to evaluate the sensitivity of the primary outcome to missing data. All possible combinations of assumed outcomes (event vs. no event) for participants with missing data in each arm will be explored. The treatment effect will be re-estimated under each scenario, and the results will be summarized to identify combinations where the statistical conclusion changes. This analysis will illustrate how extreme the assumptions about missing data must be to alter the primary findings.

To maximize the use of available outcome data, participants with missing covariate values will be retained in the primary analysis using imputation methods. Given that missingness in covariates is generally not expected to bias treatment effect estimates, a simple imputation by mean

substitution for continuous variables and mode substitution for categorical variables will be applied.

8.1.4 Multiple comparisons

Adjustment for multiplicity is applied to account for interim analyses, using the Lan-DeMets alpha spending function approach with O'Brien-Fleming-like boundaries. This method controls the overall Type I error rate by allocating a smaller portion of the alpha early in the trial, thereby requiring strong evidence to justify early stopping and preserving the pre-specified significance level across all analyses. Multiplicity is not a concern for the other endpoints with a descriptive interpretation.

8.2 Secondary endpoints

Secondary binary endpoints will be analysed with the same analysis method used for the primary endpoint.

The number of hospital-free days will be analysed as an ordinal outcome with death considered as the worst possible measure, i.e., -1. An analysis approach similar to that used for the primary endpoint will be employed, incorporating a logit link function to account for the ordinal nature of the outcome, while adjusting for clustering and time effects inherent in the stepped wedge design. For time-to-event endpoints (time to neonatal mortality and time to culture-positive sepsis), no formal statistical tests will be performed. However, log-rank tests will be reported for exploratory purposes along with the Kaplan-Meier curves. Participants with missing data on the primary outcome will be excluded from the analysis of all secondary endpoints. As all secondary endpoints are exploratory and will not undergo statistical significance testing, no formal adjustment for multiple comparisons will be made.

8.3 Exploratory endpoints

Exploratory endpoints will be analysed descriptively with means and standard deviations for continuous variables, and proportions for categorical variables. Additionally, graphical summaries will be provided.

9. Interim Analysis and Trial Stopping Guidance

Two interim analyses will be performed on the primary and secondary outcomes after the 4th and 7th sites have been randomized and have completed their three-month intervention period to

assess the safety of the study. An independent statistician will perform the interim analyses. The statistician will report to the DSMB, who will have unblinded access to the data. The DSMB will advise the investigators and other stakeholders if, based on the interim analysis results and the pre-specified adaptive design, a definitive conclusion has been reached regarding the active intervention compared to the control (standard management). The DSMB will discuss the interim analysis results with the investigators in a blinded fashion in a joint meeting. The DSMB will make a recommendation on trial continuation or early stopping based on the results of the interim analyses and pre-specified stopping rules. The DSMB can advise the investigators to stop the study for early success if significant benefits are observed in the intervention arm at an interim analysis. The study sponsor will inform the Institute Ethics Committee of the DSMB's decisions. The early stopping thresholds, assuming information fractions of 38% and 75%, are provided in Table 2. The corresponding minimum observed relative risk across all simulation scenarios that stop for futility is 1.03 at interim 1 and 0.81 at interim 2.

Table 2: Early stopping thresholds

Analysis	Information Fraction	Nominal p-value Threshold for Futility	Nominal p-value Threshold for Success
Interim 1	38%	0.6623	0.0006
Interim 2	75%	0.1568	0.0098
Final	100%	0.0218	0.0218

10. Safety Monitoring

No significant harm is anticipated due to the intervention. No specific adverse events will be reported as part of the study results. During the study's interim analyses, data on the primary outcomes of the study -mortality and/or culture-positive sepsis- will be supplied to the DSMB, together with any other required analyses, in strict confidence. Given that these outcomes are serious events, DSMB can decide to stop the trial early if there are concerns about a significantly higher incidence of mortality and/or culture-positive sepsis in the intervention arm compared to the control arm.
